# Convergent Transcription Induces Dynamic DNA Methylation at *disiRNA* Loci

**DOI:** 10.1371/journal.pgen.1003761

**Published:** 2013-09-05

**Authors:** Yunkun Dang, Liande Li, Wei Guo, Zhihong Xue, Yi Liu

**Affiliations:** Department of Physiology, The University of Texas Southwestern Medical Center, Dallas, Texas, United States of America; University of California Riverside, United States of America

## Abstract

Cytosine methylation of DNA is an important epigenetic gene silencing mechanism in plants, fungi, and animals. In the filamentous fungus *Neurospora crassa*, nearly all known DNA methylations occur in transposon relics and repetitive sequences, and DNA methylation does not depend on the canonical RNAi pathway. disiRNAs are Dicer-independent small non-coding RNAs that arise from gene-rich part of the *Neurospora* genome. Here we describe a new type of DNA methylation that is associated with the *disiRNA* loci. Unlike the known DNA methylation in *Neurospora*, *disiRNA* loci DNA methylation (DLDM) is highly dynamic and is regulated by an on/off mechanism. Some disiRNA production appears to rely on pol II directed transcription. Importantly, DLDM is triggered by convergent transcription and enriched in promoter regions. Together, our results establish a new mechanism that triggers DNA methylation.

## Author Summary

DNA methylation in eukayrotes refers to the modification of cytidines at 5^th^ position with methyl group (5mC). Though absent in some species, DNA methylation is conserved across fungi, plants and animals and plays a critical role in X chromosome inactivation, genomic imprinting, transposon silencing etc. In addition, DNA methylation also occurs at the promoter sequence to regulate gene expression. Filamentous fungus *Neurospora crassa* has a well-known mechanism of DNA methylation for genomic defense. During sexual stage repetitive sequences (e.g. transposons) are recognized and point mutations are introduced. During vegetative stage these mutations serve as signals for establishing static DNA methylation to silence all copies of the sequences. In this study, we report a new type of DNA methylation in *Neurospora*. It is tightly linked to a type of non-coding small RNA termed dicer-independent siRNA (disiRNA) and therefore was termed *disiRNA* loci DNA methylation (DLDM). DLDM is dynamic regulated and shows an on/off pattern, i.e. most alleles contain no 5mC but some are densely methylated. Interestingly, DLDM can be triggered by convergent transcription and is accumulated at promoter regions. In summary, our findings demonstrate a new type of dynamic DNA methylation.

## Introduction

DNA methylation at the 5th position of cytosine to form 5-methylcytosine (5mC) is an important epigenetic gene silencing mechanism conserved from plants, fungi to animals [1], [2]. Even though most of the DNA methylation is relatively stable, dynamic DNA methylation has been observed during specific stages of animal development [3]. DNA methylation occurs in three different nucleotide sequence contexts: CG, CHG, and CHH (where H is C, A, or T). Small non-coding RNAs have been shown to be involved in the establishment and maintenance of heterochromatin formation in different organisms. In the fission yeast *Schizosaccharomyces pombe*, small RNAs and the RNAi pathway mediate histone H3 lysine-9 methylation at the centromeric regions [4]–[6]. In plants, the asymmetrical CHH methylation is maintained by *de novo* DNA methylation mediated by 24-nt small interfering RNAs (siRNAs) [review in 7], [8]. In mammalian germ cells, the Dicer-independent Piwi-interacting RNAs (piRNAs) are thought to be involved in DNA methylation [9], [10].

In the filamentous fungus *Neurospora crassa*, about 2% of cytosines in the genome are methylated [11]. Nearly all of the known methylation sites are within transposon relics of repeat-induced point mutation (RIP) and the repetitive ribosomal DNA locus [11]–[13]. RIP is a genome defense mechanism that results in mutation and methylation of duplicated DNA sequences during the sexual cycle [11], [14]. All previously known *Neurospora* DNA methylation is dependent on the histone methyltransferase, DIM-5, which meditates trimethylation of histone H3 at the lysine9 (H3K9me3) [15], [16]. Heterochromatin protein 1 (HP1) recognizes H3K9me3 and recruits DIM-2, the only confirmed DNA methyltransferase in *Neurospora*, to methylate DNA [17]–[19]. For the natural RIP'd sequences, DNA methylation is more or less stable and is generally not required for the maintenance of H3K9 methylation [12]. In addition, the known DNA methylation events are not dependent on the canonical RNAi pathway [20].

*Neurospora* produces many types of small RNAs, including microRNAs, siRNAs, QDE-2 interacting RNA (qiRNAs), and dicer-independent siRNAs (disiRNAs), through diverse small RNA biogenesis pathways [reviewed in 21]. disiRNAs are a distinct class of small RNAs as they symmetrically are mapped to both strands of DNA and their production is independent of the known canonical RNAi components, including Dicer [22]. The *disiRNA* loci range from a few hundred base pairs to more than 10 kilobases in size and are located in gene-rich regions of the genome. The function of disiRNA is unknown.

In this study we identified a new mechanism of DNA methylation that is associated with *disiRNA* loci. Our results showed that this type of DNA methylation, which we call *disiRNA* loci DNA methylation (DLDM), is very different from the previously known DNA methylation in *Neurospora*. DLDM is highly dynamic, depends on transcription at *disiRNA* loci, and is triggered by convergent transcription in some loci.

## Results

### DNA methylation at the disiRNA loci

**Figure 1:**
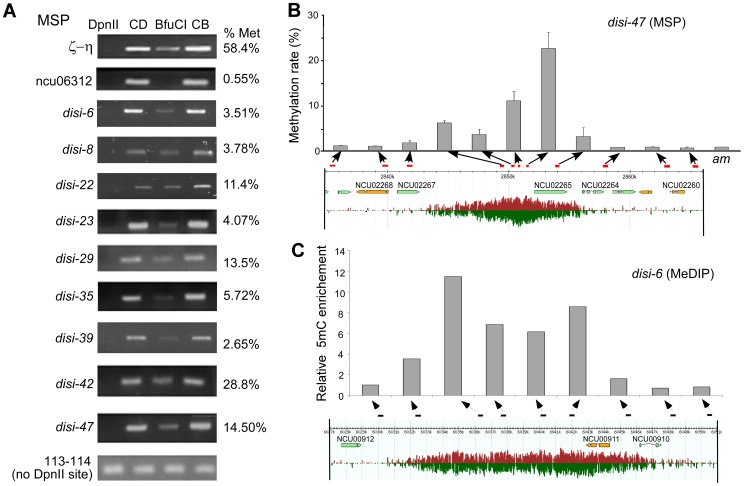
*disiRNA* loci are methylated. (A) Methylation-specific PCR (MSP) analyses using *Dpn*II or *Bfu*CI showing that *disiRNA* loci are methylated. CD and CB are samples that were only treated with either *Dpn*II or *Bfu*CI restriction digestion buffer, respectively. *ζ-η* and *ncu06312* gene regions were used as positive (methylated) and negative (unmethylated) controls, respectively. Primer pair 113–114 covers a region without DpnII/BfuCI recognition sites. Agarose gel images show the results of semi-quantitaive PCR analyses. The estimated percentages of methylation (right) for these loci were determined by qPCR. (B) Correlation between DNA methylation level (upper panel) and disiRNA expression profile (low panel) spanning the *disi-47* locus as determined by MSP using qPCR. The *am* locus served as a negative control. (C) Correlation between DNA methylation level and disiRNA expression profile at the *disi-6* locus determined by MeDIP. Arrows indicate the locations of primer pairs (see Figure S1 and Table S1 for primer information).

DNA methylation is an important regulatory mechanism of transcription silencing. Therefore, we examined whether in *Neurospora* the *disiRNA* loci are associated with DNA methylation using the methylation-sensitive restriction enzyme-based PCR (MSP) assays [23]. The *Neurospora* genomic DNA from a wild-type strain was digested with the isoschizomers *Dpn*II or *Bfu*CI. Both enzymes digest unmethylated GATC sites, but only *Dpn*II can cut at sites when C is methylated. Primer sets were then used for semi-quantitative and quantitative PCR (qPCR). As shown in Figure 1A, a non-*disiRNA* locus (NCU06312) was not methylated, as indicated by the lack of PCR amplification product after digestion by *Bfu*CI, whereas a PCR product was readily detected for the *ζ-η* region, a relic of RIP that forms constitutive heterochromatin and was previously shown to carry DNA methylation [14], [24]. For the 9 selected *disiRNA* loci with high level of disiRNA and with size larger than 5 kb, PCR products were detected in all samples after *Bfu*CI digestion, indicating that these *disiRNA* loci are methylated. From quantitative PCR (qPCR) analyses, we estimated that percentages of methylation at the *Dpn*II sites in these loci ranged from ∼3.5% to 28.8%, levels much lower than that of the *ζ-η* region (58.4%).

We then applied qPCR to MSP to further determine the DNA methylation profile in *disi-47*, *disi-6*, and *disi-29* loci, which are three large loci with high levels of disiRNAs (Figure S1). The DNA methylation profiles at these loci are correlated with disiRNA profiles: DNA methylation peaked at regions of peaked disiRNA expression, and there was little or no DNA methylation outside of the *disiRNA* loci (Figure 1B and Figure S2).

Since the MSP results only reflect the methylation status of cytosines within the cleaved GATC sites, the detected methylation levels might be biased. To exclude this possibility, we performed methylated DNA immunoprecipitation (MeDIP) to detect DNA methylation based on 5mC density in *disiRNA* loci. For 13 loci (disi-6, 8, 22, 23, 29, 34, 35, 39, 42, 47–50) with relatively high disiRNA level and with size larger than 5 kb, all of them harbor DNA methylation (data not shown). We further analyzed *disi-6, disi-29* and *disi-47* loci with more primer sets for better resolution. As expected, the levels of DNA methylation were high in regions of high disiRNA expression and were low or absent outside of the disiRNA loci (Figure 1C and Figure S3). In constrast, no DNA methylation was detected at two negative control loci *al-1* and *NCU06312*.

Since the previously known DNA methylation in *Neurospora* is located in regions derived from RIP, we calculated the RIP indices of the methylated *disiRNA* loci [25]. We observed no significant difference in RIP indices between that of the whole genome (1.11±0.33) and all of the 50 identified *disiRNA* loci (1.13±0.32). Moreover, for each *disi-6*, *disi-29* and *disi-47* loci, the lowest RIP indices are well above the threshold (0.7) for RIP-induced DNA methylation [25] (Figure S4). These data suggest that the DNA methylation in these *disiRNA* loci is not a result of RIP. We therefore named this type of DNA methylation DLDM (disiRNA loci DNA methylation) to distinguish it from the RIP-induced DNA methylation.

### The on/off methylation pattern of DLDM

**Figure 2:**
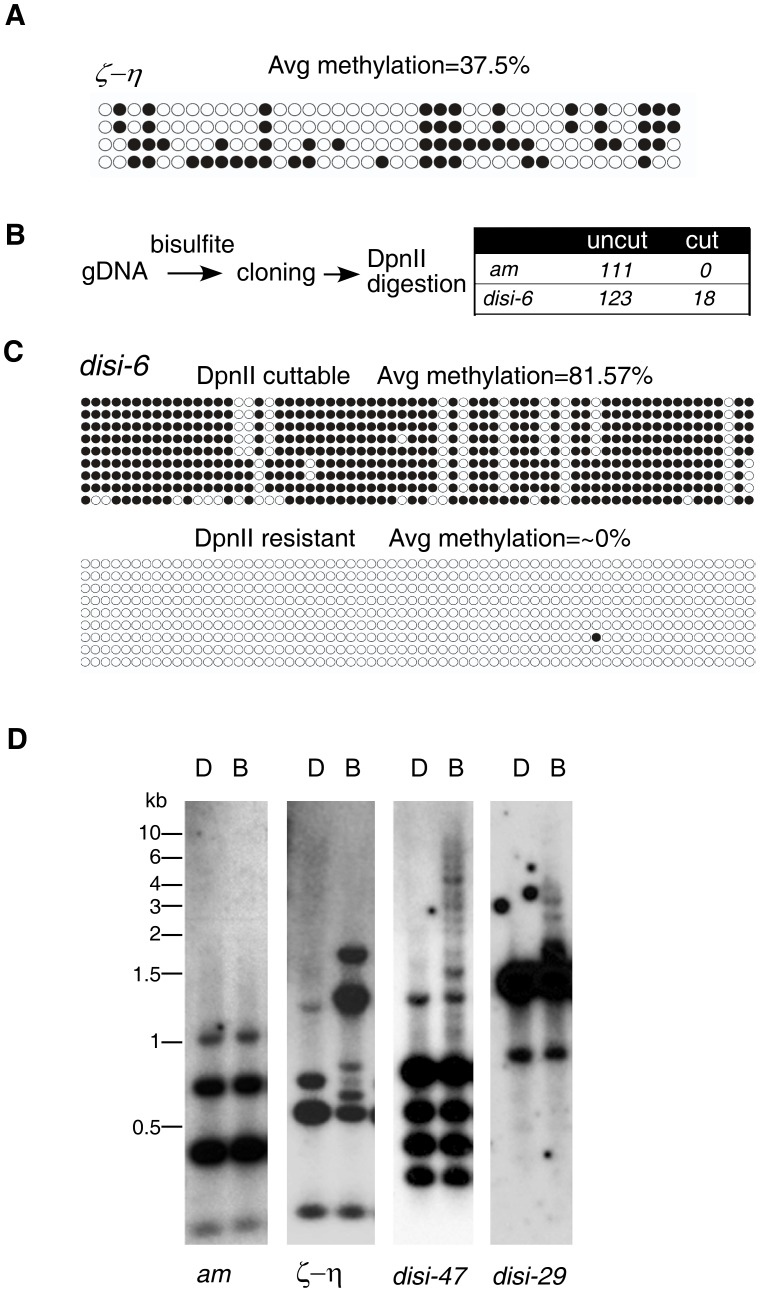
*disiRNA* loci have an on/off pattern of DNA methylation. (A) Bisulfite sequencing result of the *ζ-η* locus reveals significant methylation in all DNA clones examined. Each row of circles represents the number of cytosines in one subcloned *ζ-η* fragment. Opened and filled circles indicate unmethylated and methylated cytosines, respectively. (B) Strategy for detecting methylation in *disi-6* locus (strategy 1, Materials and Methods). The lack of cleavage by *Dpn*II indicates no methylation at the GATC recognition site of the original sequence, whereas the cleavage by *Dpn*II indicates that the DNA sequence contained 5mC. (C) 9 clones from “cut” and “uncut” populations were subject to sequencing. DNA methylation profiles were shown. (D) Southern blot analyses of *disi-29* and *disi-47* loci. The *am* and *ζ-η* loci were as the negative and positive control, respectively.

To determine the nature of DLDM, we performed bisulfite sequencing of selected genomic regions. As controls, we first sequenced the *ζ-η* region, a relic of RIP process and the *am* locus, which was previously shown to be unmethylated [26]. As expected, every clone of the DNA at the *ζ-η* region was methylated to various degrees at both symmetric and non-symmetric cytosine sites with an average methylation frequency of 36% (Figure 2A), similar to the estimated frequency (42%) determined in the MSP assay. In contrast, the genomic DNA was unmethylated at the *am* locus. In our experiment, 99.5% of all cytosines were converted to uracils. This percentage was similar to the conversion rate (99.8%) of an unmethylated PCR fragment after the bisulfite treatment, indicating that our bisulfite conversion was complete (data not shown). To our surprise, bisulfite sequencing of the *disiRNA* regions with peak DNA methylation revealed that vast majority of the clones were not methylated, as indicated by the nearly 100% conversion of all cytidines into uracils. For a few of the *disiRNA* loci clones, however, almost all cytosines were maintained after the bisulfite treatment (data not shown).

To rule out the possibility that these highly methylated clones were due to incomplete bisulfite conversion, we first treated the genomic DNA with bisulfite, then performed PCR and subcloned the fragments of either the *disi-6* or the *am* locus into plasmids. PCR was then performed by using the plasmids as templates, and the resulting PCR products were subjected to *Dpn*II digestion, which can only digest the PCR products if GATC site remains intact after bisulfite conversion due to the protection of 5mC (Figure 2B). As expected, all 111 clones of DNA from the *am* locus examined were resistant to the *Dpn*II digestion, indicating that the bisulfite treatment of the genomic DNA was complete. For the *disi-6* locus, however, 18 out of 123 DNA clones were cleaved by *Dpn*II. Sequencing of the *Dpn*II digestible (“cut”) and resistant (“noncut”) clones of *disi-6* locus showed that in all “noncut” clones nearly 100% of cytosines were converted to uracils, indicating that the initial genomic DNA carried no 5mC (Figure 2C). In contrast, most of the cytosines in the *Dpn*II digestible clones were methylated. Similar results were obtained from the *disi-47* and *disi-29* loci (Figure S5). Since *Neurospora* is haploid, these results indicate that the DNA methylation at *disiRNA* loci is actively regulated and is either on or off within each nucleus: *disiRNA* loci are either not methylated or are heavily methylated once DNA methylation process is switched on.

To confirm this finding, we used Southern blot analysis to visualize DNA methylation at several *disiRNA* loci. As shown in Figure 2D, both *Dpn*II and *Bfu*CI resulted in the same digestion pattern of the *am* locus, consistent with the lack of DNA methylation. For the *ζ-η* region, the *Bfu*CI digestion resulted in the disappearance of one DNA fragment and the appearance of several additional higher molecular weight DNA fragments, consistent with all DNA molecules from this locus being methylated to some degree. The largest DNA fragment from *Bfu*CI digestion was less than 2 kilobases (kb), indicating that methylation in the *ζ-η* region is limited to a small region. For the *disi-47* and *disi-29* loci, however, *Bfu*CI digestion did not significantly change the levels or the relative ratios of the *Dpn*II-digested bands, suggesting that most of the DNA lack methylation. We did observe, however, a ladder of high molecular weight (up to 8–10 kb) DNA fragments in the *Bfu*CI-digested DNA, indicating that when methylated, the DNA at the *disiRNA* loci are heavily methylated across a large DNA region. Taken together, these results suggest that DLDM is highly dynamic and is regulated differently from the known DNA methylation events within relics of RIP in *Neurospora*.

### Methylation of H3K9 at the disiRNA loci is dependent on DIM-2

**Figure 3:**
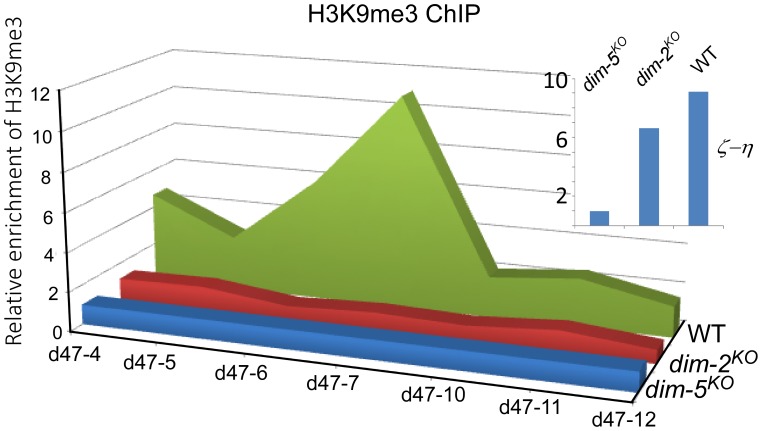
Histone H3K9me3 modification in the *disiRNA* loci is dependent on DIM-2. The distribution of the H3K9me3 modification in wild type, *dim-2^KO^* and *dim-5^KO^* mutants at the *disi-47* locus was determined by ChIP using an H3K9me3 antibody. Relative enrichment of DNA was calculated by normalizing with the relative DNA binding levels of the *dim-5^KO^* mutant. The insert panel shows H3K9me3 ChIP assay results at the *ζ-η* region in the indicated strains.

The histone modification of H3K9me3 is mediated by the H3K9 methyltransferase DIM-5. This enzyme is essential for DNA methylation at relics of RIP, but H3K9 methylation at these loci is generally maintained in the absence of DNA methylation [12], [27]. To determine whether DLDM is mediated by H3K9me3 modification, we performed H3K9me3 chromatin immunoprecipitaton (ChIP) assays. Our results showed that the *disiRNA* loci are enriched by histones containing the H3K9me3 mark (Figure 3 and S6). However, in contrast to the *ζ-η* locus, in which the levels of H3K9me3 were not affected by the deletion of *dim-2^KO^*, the levels of H3K9me3 at the *disi-47, -6 and -29* loci decreased dramatically in the *dim-2^KO^* mutant. These results indicate that DLDM requires DIM-2 for the maintenance of H3K9me3 at the *disiRNA* loci.

### DLDM is linked with convergent transcription and disiRNA

As *disiRNA* loci are gene rich, we examined whether transcription was important for disiRNA and DLDM. Our previous EST analyses showed that at least some *disiRNA* loci harbor fully or partially overlapped antisense transcripts, suggesting that convergent transcription might be a trigger of disiRNA production and/or DLDM [22]. We chose *disi-47* locus to test this hypothesis since it harbors the well-studied circadian clock gene *frequency* (*frq, NCU02265*), which is known to produce the sense *frq* transcript and an overlapping antisense transcript *qrf* [28], [29]. The *frq* gene encodes a core component of the *Neurospora* circadian clock. MeDIP and MSP assays showed that the promoter region of the *frq* gene was methylated; however, only low levels of DNA methylation were detected in the *frq* coding region (Figure S3).

**Figure 4:**
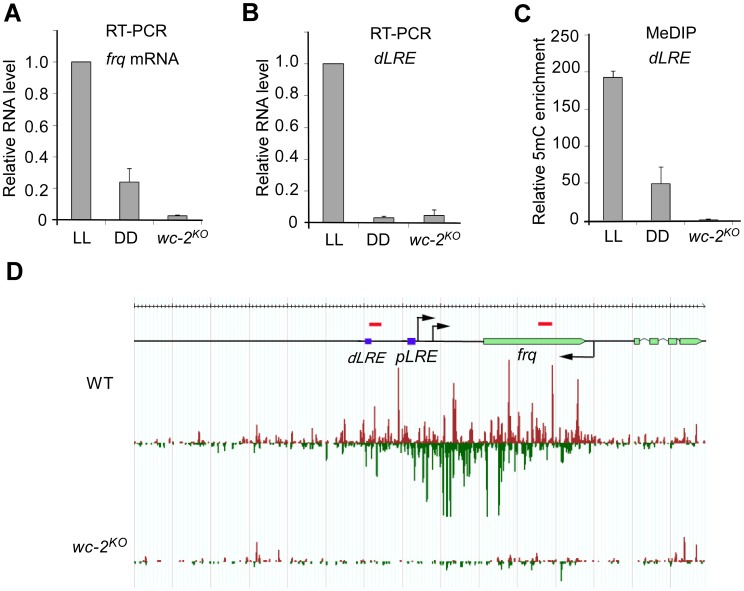
DNA methylation and disiRNA in *disi-47* locus requires transcription. (A) The disiRNA distribution at *disi-47* locus in wild-type and *wc-2^KO^* strain. Arrows indicate the transcription start sites. dLRE and pLRE are the two WC complex binding sites. The red bars indicate approximate primer set locations (see Table S1 for primer sequences). (B) RT-qPCR analyses of *frq* mRNA. (C) RT-qPCR analyses of the transcripts in the *frq* promoter region from a wild-type strain grown in constant light (LL) or constant darkness (DD) conditions and the *wc-2^KO^* strain grown in LL. (D) MeDIP results of the dLRE region in the wild-type (LL and DD conditions) and the *wc-2^KO^* strains (LL). In (B), (C) and (D), three independent repeats were performed. Values are mean ± s.d.

The transcription of both *frq* and *qrf* is activated by light in a process that mediated by the WHITE COLLAR (WC) complex, which consists of two PAS domain-containing transcription factors WC-1 and WC-2 [29]. Previous studies showed that the expression of both *frq* and *qrf* are high in constant light (LL) and are low in constant darkness (DD) [28]. Our qRT-PCR assays also confirmed that the level of *frq* mRNA was significant higher in LL than in DD (Figures 4A). Importantly, our mRNA deep sequencing [30] and RT-qPCR assays also demonstrated the existence of light-dependent expression of transcripts from the promoter region of *frq* (Figure 4B and S7), where disiRNA level is high. In a *wc-2^KO^* mutant, *frq* mRNA and the transcripts originating from the promoter region were both abolished, indicating that, like *frq* mRNA, the promoter-specific transcription requires WC-2. MeDIP assays showed that the level of DNA methylation in the promoter of *frq* was significantly higher in LL than that in DD in the wild-type strain and was completely abolished in the *wc-2^KO^* mutant (Figure 4C). Together, these results suggest that DNA methylation at the *frq* promoter is dependent on WC-2-mediated transcription and that transcription at promoter region may be necessary for the DNA methylation process and disiRNA production. Similarly, divergent promoter transcripts are also clearly seen in other disiRNA loci such as *disi-29* (Figure S7), suggesting that this architecture of transcription might be the reason of DLDM.

To determine whether disiRNA production is dependent on the WC-dependent transcription, we performed small RNA deep sequencing analyses in *wc-2^KO^* strain. As shown in Figure 4D, the disiRNA abundance in the mutant is completely abolished at the *disi-47* locus. This result is consistent with the loss of DLDM in the *wc-2^KO^* strain (Figure 4C) and suggests that disiRNA and DLDM are tightly linked and both triggered by pol II-dependent transcription.

#### Convergent transcription triggers DNA methylation in the promoter region

**Figure 5:**
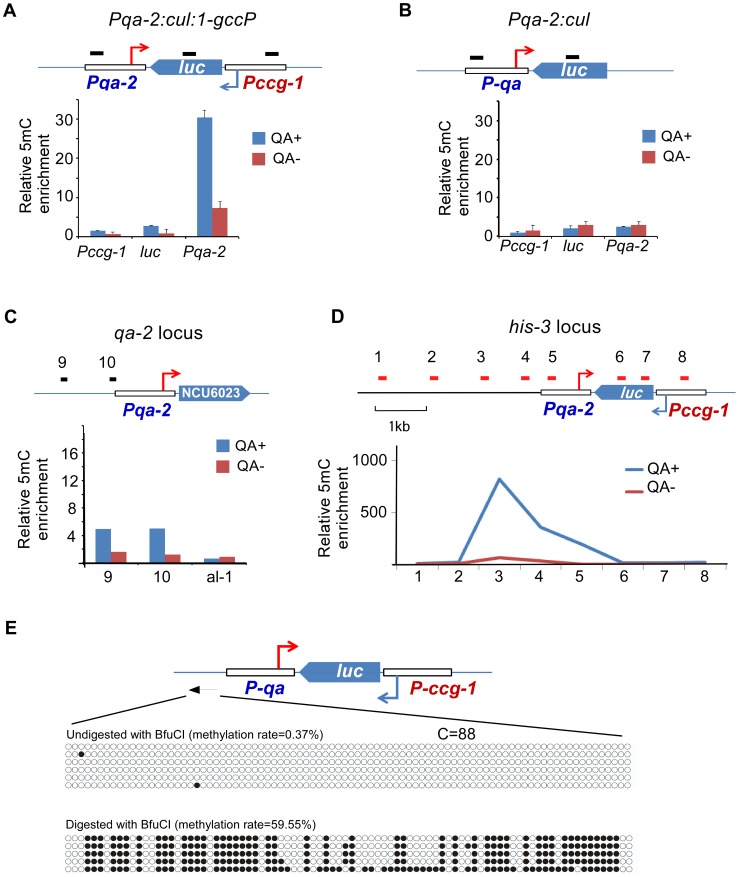
DNA methylation is induced in the promoter region of an artificial convergent transcription construct upon induction of transcription. (A and B) MeDIP results showing the DNA methylation status of the *ccg-1* promoter, *luc* gene body, and the *qa-2* promoter of the indicated construct. The artificial construct *Pqa-2:cul:1-gccP* (A) or *Pqa-2:cul* (B) resides at the *his-3* locus. The top cartoon of each panel depicts the architecture of the construct and black bars indicate the approximate location of primer sets. Three independent repeats were performed. Values are mean ± s.d. (C) Distribution of DNA methylation at the endogenous location of the *qa-2* promoter of the *Pqa-2:cul:1-gccP* strain. (D) Distribution of DNA methylation around the *Pqa-2:cul:1-gccP* construct at the *his-3* locus with/without the activation of the *qa-2* promoter. In panels A-D, QA+ and QA- indicate presence or absence of quinic acid (QA), respectively. DNA methylation was measured with MeDIP. (E) Results of bisulfite PCR in the region upstream of the *qa-2* promoter of the *Pqa-2:cul:1-gccP* construct. Two aliquots of genomic DNA from *Pqa-2:cul:1-gccP* strain, one digested with *Bfu*CI and one untreated, were subject to bisulfite treatment and sequencing, respectively (strategy 2 of bisulfite sequencing, primer sequences in table S4). Each row of circles represents the order and number of cytosines in the subcloned sequence. Opened and filled circles indicate unmethylated and methylated cytosine, respectively.

To further test the possibility that convergent transcription is a trigger of DLDM, we created an artificial sense-antisense transcription construct *Pqa-2:cul:1-gccP*, in which a firefly luciferase (*luc*) gene is under the control of *ccg-1* promoter and the quinic acid (QA) inducible promoter is used to drive the antisense transcription of the *luc* gene (Figure 5A). The construct was introduced into a *dicer* double mutant so that the convergent transcripts cannot be processed into siRNA from dsRNA [31]. As a control, a strain that contains a construct (*Pqa-2:cul*) lacking the *ccg-1* promoter was analyzed (Figure 5B). MeDIP analyses showed that the addition of QA to growth medium resulted in a robust induction of DNA methylation in the promoter region of *qa-2* in the *Pqa-2:cul:1-gccP* strain (Figure 5A). In contrast, no methylation of the *qa-2* promoter was detected in the *Pqa-2:cul* strain with or without QA treatment (Figure 5B). In addition, the promoter of the endogenous *qa-2* was also not methylated in a wild-type strain after QA treatment (Figure 5C). These results indicate that convergent transcription induces DNA methylation that peaks in the promoter region.

To determine the size of the methylated region in the transgene region in the *Pqa-2:cul:1-gccP* strain, we performed MeDIP analyses using primer sets spanning the transgene locus. As shown in Figure 5D and S8A, only low levels of DNA methylation were detected in the absence of QA; these levels are likely due to the leakage of the *qa-2* promoter. The presence of QA resulted in a dramatic induction of DNA methylation. The peak of DNA methylation was upstream of the *qa-2* promoter and about 3 kb upstream of the predicted *qa-2* transcriptional start site (TSS). Only low levels of DNA methylation were detected in the *luc* gene and in the *ccg-1* promoter. Interestingly, the peak of the DNA methylation in the *disi-47* locus was also about 3 kb upstream of the known *frq* transcription start site (Figure S3). These results indicate that the transcription-induced DLDM can spread several kb upstream of the gene promoter.

To determine whether the DNA methylation induced in the artificial convergent transcription construct also showed an on/off switch pattern as observed in the *disiRNA* loci, we performed bisulfite sequencing. Similar to *disiRNA* loci, there was almost no methylated cytosine in most of the randomly selected PCR clones from genomic DNA, indicating that most of the DNA molecules were not methylated. In contrast, when the genomic DNA was first treated with *BfuC*I to enrich for the methylated alleles, we observed that most of cytosines upstream of the *qa-2* promoter were methylated (Figure 5E). These results suggest that convergent transcription triggers dynamic DNA methylation in the *disiRNA* loci. In addition, in sRNA deep sequencing analyses, we observed sRNA accumulation in the upstream region of *qa-2* promoter at the *his-3* locus that contains *Pqa-2:cul:1-gccP* construct but not in that of the endogenous *qa-2* locus upon QA induction (Figure S8B and S8C), indicating a link between DLDM and disiRNA production. The dependence of DLDM on transcription also provides an explanation for the on/off pattern of DLDM because DLDM may require a certain threshold of transcription from the *disiRNA* loci.

## Discussion

*Neurospora* is a well-established model system for DNA methylation. All previously known DNA methylation in *Neurospora* occurs in relics of RIP [12], [32]. RIP is a process that silences repetitive DNA sequences during sexual stage (prior to meiosis) by converting cytosine to thymine in target sequences and occurs mostly at CpA dinucleotide context [33]. The resulted A/T rich region then serves as a signal that induces methylation of the nearby region to silence gene expression. In this study, we showed that DLDM is established and maintained very differently from the RIP-induced DNA methylation.

First, DLDM occurs in the gene-rich *disiRNA* loci that contain no relics of RIP or other repetitive elements. Second, in contrast to RIP'd regions in which DNA methylation is more or less constitutive and occurs in all alleles, most of the alleles are not methylated at *disiRNA* loci. In *disiRNA* loci, only a small percentage of alleles are extensively methylated with most of cytosines modified over a region that extends several kilobases. The dense cytosine methylation is similar to recently demonstrated dense methylation/hydroxylmethylation of cytosines in mouse embryonic stem cells [34]. The on-off pattern of DLDM indicates that DLDM is highly dynamic and that there is an inducible mechanism that mediates the establishment of DLDM. On the other hand, a de-methylation process may also exist to convert methylated alleles back into unmethylated alleles. Third, unlike the DNA methylation in the RIP'd regions, which is generally not required for maintenance of H3K9 methylation, DLDM is required for the maintenance of H3K9 methylation at the *disiRNA* loci. It suggests that an unknown mechanism should exist to recognize DNA methylation and in turn trigger H3K9me3.

Finally, DLDM is dependent on transcription. We demonstrated that DLDM is induced by convergent transcription from artificial constructs expressed in *Neurospora*. This conclusion is in agreement with the fact that most of disiRNA loci are known to produce sense and antisense RNAs [22]. Interestingly, the peaks of DNA methylation occurred at the promoter regions, where disiRNA expression also peaked. In addition, promoter-specific RNA transcripts were detected, and levels of these transcripts correlated with the levels of DNA methylation, suggesting that these non-coding RNA transcripts are involved in DLDM and are the precursors for disiRNAs. The induction of DLDM by transcription may explain its on/off pattern and suggests that a certain threshold level of transcription may be required for the establishment of DNA methylation. These results indicate that DLDM differs substantially from the typical DNA methylation in RIP'd DNA regions. It should be noted that transient DNA methylation was recently reported at the *frq* locus and was shown to be involved in setting the proper phase of circadian clock during the preparation of this paper [35]. In addition, the distribution of DNA methylation induced by convergent transcription is mainly accumulated upstream and peaks at about 3 kb from the TSS, suggesting that DLDM might also be involved in suppressing the promiscuous transcription at promoter region during transcriptional initiation. Indeed, recent studies suggest that pol II-directed gene transcription may adopt a gene loop structure by tethering promoter and terminator sequence, which enhances the transcriptional directionality toward the gene body [36]–[38]. Therefore, it is possible that the DNA methylation is a result of complex interaction between both ends of the gene for transcription initiation and termination, which strengthens the directionality of both sense and antisense transcription.

## Materials and Methods

### Strains and culture conditions

In this study, FGSC 4200 (a) was used as wild-type (WT) strain. Mutant strains *wc-2^KO^*, *qde-1^KO^*, *qde-2^RIP^*, *qde-2^RIP^;sms-2^RIP^* double mutant, and *dcl-1^RIP^;dcl-2^KO^* double mutant (*dcl^DKO^*) were generated in previous studies [31], [39], [40]. The *dim-2^KO^* and *dim-5^KO^* strains were generously provided by Dr. Qun He [23]. Liquid cultures were grown in minimal medium (1× Vogel's, 2% glucose) at 30°C overnight and then at room temperature with shaking at 130 r.p.m. for 24 h [41]. For liquid cultures containing QA, 0.01 M QA, pH 5.8, was added to the liquid culture medium containing 1× Vogel's, 0.1% glucose and 0.17% arginine.

To make the *his-3* targeting *Pqa-2:cul:1-gccP* constructs, a PCR fragment containing the promoter of *ccg-1* was inserted into the plasmid pDE3dBH-Pqa-2 [42] to generate *Pqa-2::1-ccgP*. Then a PCR fragment of luciferase gene (*luc*) was inserted between the two promoters, with the *luc* sense transcripts and antisense transcripts driven by *ccg-1* and *qa-2* promoter, respectively. The control construct, *Pqa-2:cul*, was created by inserting the *luc* gene into pDE3dBH-Pqa-2, with *qa-2* promoter driven antisense transcription of *luc*. The resulting constructs were introduced into the *his-3* locus of *dcl^DKO^*, *his*-*3* strain [39], a *his-3* strain and an *eri-1l^KO^*, *his-3* recipient strain.

### Southern blot analyses

Approximately 10 µg genomic DNA was digested with *Bfu*CI or *Dpn*II, fractioned in 1.0% agarose gels, and transferred to nylon membrane. Hybridization probes were prepared from PCR products of interest (primer sequences in Table S1) with Rediprime II DNA Labeling System (GE Healthcare).

### Methylated DNA immunoprecipitation (MeDIP)

Approximately 10 µg genomic DNA was sonicated into small fragments (size ∼300–1000 bp). In each reaction, 1 µg of the 5-methylcytosine monoclonal antibody (Epigentek) was used to perform the MeDIP assay as previously described [12], [43]. MeDIP samples were analyzed with qPCR with corresponding primers listed in Table S1. In order to compare methylation in different regions, relative enrichment of DNA was calculated as the ratio of MeDIP sample over its input (set as 1), and the qPCR result of a primer pair of the *am* locus was used for normalization to correct for possible primer efficiency bias [44]. To compare MeDIP results of different samples or treatments, we performed the MeDIP at the same time with same batch of anti-5mC antibodies, due to the variation of MeDIP efficiency for different batches of antibodies.

### Chromatin immunoprecipitation assay

The ChIP assay was performed as previously described [45]. The immunoprecipitation was performed with an H3K9me3 antibody (Abcam ab8898). The relative enrichment was calculated as the MeDIP assay and the qPCR result of a primer pair of the *am* locus was used for normalization.

### Methylation specific PCR and qPCR

Methylation specific PCR (MSP) was performed as previously described [23]. The methylation rate, determined by quantitative PCR, was calculated as the ratio of BfuCI-digested DNA signal to its input. A primer pair for (113–114), whose PCR product carries no BfuCI/DpnII recognition site (GATC), was used to normalize for loading and primer efficiency.

### Bisulfite PCR methylation analysis

The bisulfite PCR methylation analysis was carried out in three steps: 1) The bisulfite treatment of genomic DNA was carried out as described in the manual of EpiTech Bisulfite Kit (Qiagen) except that we used a modified thermal cycler condition: 99°C for 5 min followed by 60°C for 25 min; 99°C for 5 min followed by 60°C for 85 min repeated 3 times; and 99°C for 5 min followed by 60°C for 90 min. 2) Two rounds of nested PCR were performed; the PCR product of first round was diluted 10–100 fold and 1 µL was used for second round of PCR. The second round PCR products of the expected size were cloned into TOPO clone kit (Invitrogen) and individual clones were sequenced.

Two strategies were used to examine DNA methylation in *disiRNA* loci. Strategy 1, shown in Figure 2, used plasmids as templates to amplify the cloned fragment. The genomic counterpart of the cloned fragment carries a GATC site. If the site was methylated, the fragment would be resistant to bisulfite treatment, whereas the unmethylated sites were converted into uridine and no longer recognized by *Dpn*II or *Bfu*CI. By identifying whether the PCR product was resistant to *Dpn*II or not, we could distinguish whether the cloned fragment was methylated in the GATC sequence. Strategy 2, used in experiments in Figure S5, is similar to the first strategy except that one aliquot of genomic DNA was treated and one was not treated with *Bfu*CI before bisulfite treatment, PCR, cloning, and sequencing. The average methylation rate was calculated by dividing total number of 5-methylcytosines by the total number of cytosines in the amplified sequence. The primers used for bisulfite sequencing are shown in Table S2 and S3.

### RT-qPCR

Total RNA was extracted with TRIzol (Invitrogen), digested with Turbo DNase (Ambion) and reverse transcribed into cDNA with SuperScript II (Invitrogen). β-tubulin transcripts (primer pair tub) were used as loading control for quantitative PCR.

### Small RNA sequencing analyses

Total RNA of *wc-2^KO^* strain, wild-type strain and *dicer^DKO^ Pqa-2:cul:1-gccP* strain were extracted with the TRIzol reagent (Invitrogen) and small RNAs were enriched with 5% polyethylene glycol (MW8000) and 500 mM NaCl as previously described [31]. Library construction and small RNA sequencing was performed by the Beijing Genomic Institute (Shenzhen, China) with Illumina standard protocol. All small RNA analyses were performed as described previously [22] except that an alignment tool Bowtie (ver 0.12.7) was used to map the small RNAs onto the *N. crassa* genome. In order to compare the density of small RNAs between samples, a standard normalization method was applied by scaling total reads of different samples to those of the same library size [46]–[48]. To correct bias induced by ribosomal RNA degradation products, we filtered out the reads matching rDNA regions from the total reads and used the remaining reads for scaling. The density of small RNA is presented as the relative number of small RNAs in a 100 nt non-overlapping sliding window along the Watson or Crick strand of each chromosome. The sRNA sequencing data was visualized with Generic genome browser (version 1.70) [49].

### Accession number

The NCBI accession number of the sRNA deep sequencing data reported in this study is GSE47666.

## Supporting Information

Figure S1 The disiRNA distribution *at disi-6, disi-29* and *disi-47* loci in a wild type strain. The results are based on the previous sRNA sequencing results [22]. The approximate locations of primer sets for *disi-6*, *disi-29* and *disi-47* loci were indicated with black bars. The sequences of primer sets are shown in Table S1.
(PDF)

Figure S2 The MSP results for *disi-6* and *disi-29* loci determined by quantitative PCR. A wild type strain was used.
(PDF)

Figure S3 MeDIP results for *disi-47* and *disi-29* loci of a wild type strain. The results of two negative controls (gene al-1 and NCU06312) and one positive control (*ζ-η*) are shown in the table.
(PDF)

Figure S4 RIP indices in *disiRNA* loci. RIP indices were calculated with the number of CpA and TpG dinucleotides divided by number of ApC and GpT in a 500 bp window sliding every 100 bp across *Neurospora* genome [25]. The red lines indicate the RIP index threshold for RIP'd region (0.7).
(PDF)

Figure S5 Bisulfite sequencing results for *disi-47* and *disi-29* loci. Experiments were performed with strategy 2 as described in Materials and Methods. Two aliquots of the wild-type genomic DNA, one treated with *Bfu*CI and one untreated, were subjected to bisulfite conversion and nesting PCR (primer sequences in Table S2). PCR products were subcloned, sequenced and aligned. Each circle indicates one cytidine in sequenced regions. Opened and filled circles represent unmethylated and methylated C.
(PDF)

Figure S6 H3K9me3 ChIP results of *disi-6* and *disi-29* loci. For each primer set, the ChIP results of *dim-5^KO^* strain were set as 1. The primer set at *am* locus was used as loading control.
(PDF)

Figure S7 Strand-specific mRNA high throughput sequencing results of the *disi-47* and *disi-29* loci of *wild type* strain. Blue and red dots represent the reads that match Watson strand and Crick strand, respectively. The directionality of gene and promoter transcripts is indicated with arrows. The green bars indicate the disiRNA loci on *Neurospora* genome. The mRNA sequencing sample and data analyses were prepared as previous described [30].
(PDF)

Figure S8 The DNA methylation is induced by convergent transcription and is correlated with disiRNA production. (A) An independent repeat for testing the DNA methylation triggered by convergent transcription. The experimental procedure was performed as described in Figure 5 except that a different *Neurospora* transformant was used. (B) The sRNA distribution at the endogenous *qa-2* locus and recombinant *his-3* locus. The result was from the *dicer^DKO^* strain harboring *Pqa-2:cul:1-gccP* construct upon QA induction. The red dashed boxes indicate the region upstream of the *qa-2* promoter. (C) the size distribution of sRNA at the *his-3* locus.
(PDF)

Table S1 Sequences of primer sets used for detecting methylation in *disiRNA* loci. The approximate locations of these primer sets are indicated in Figure S1.
(PDF)

Table S2 Primers used for preparing DNA probes for Southern blotting.
(PDF)

Table S3 Primers used for bisulfite sequencing of *disiRNA* loci. For each case, f1 and r1 were used for first round of PCR. The PCR product then was used to perform second round nesting PCR with f2 and r2. The am and al-1 were used as negative controls and ζ-η as positive control.
(PDF)

Table S4 Primer sets used for strains with artificial convergent transcription and control strains.
(PDF)
