# Supplementary figures and images for: Convergent Transcription Induces Dynamic DNA Methylation at disiRNA Loci
Source: PLoS Genet. 2013 Sep 5;9(9):e1003761. doi: 10.1371/journal.pgen.1003761 (PMC3764098; doi:10.1371/journal.pgen.1003761)

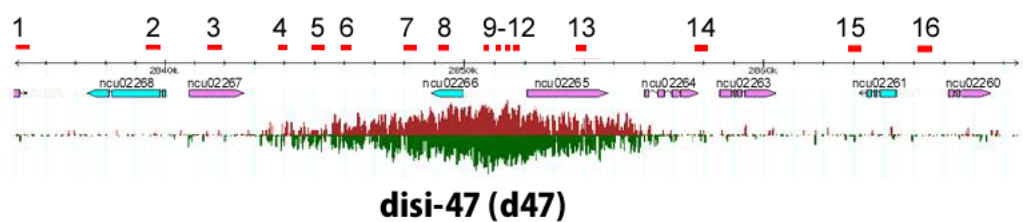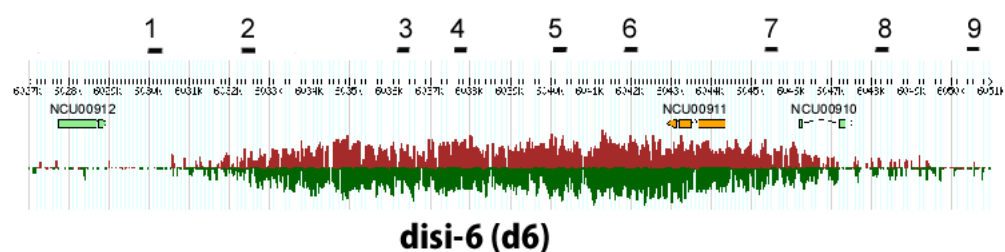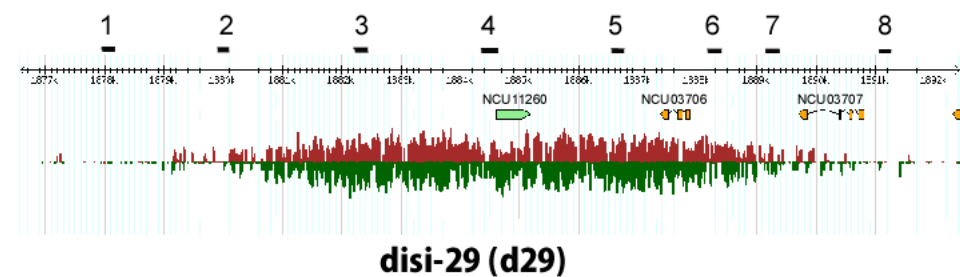

Figure S1

Supplement: Figure S1 — The disiRNA distribution at disi-6, disi-29 and disi-47 loci in a wild type strain. The results are based on the previous sRNA sequencing results [22]. The approximate locations of primer sets for disi-6, disi-29 and disi-47 loci were indicated with black bars. The sequences of primer sets are shown in Table S1. (PDF) [file pgen.1003761.s001.pdf]

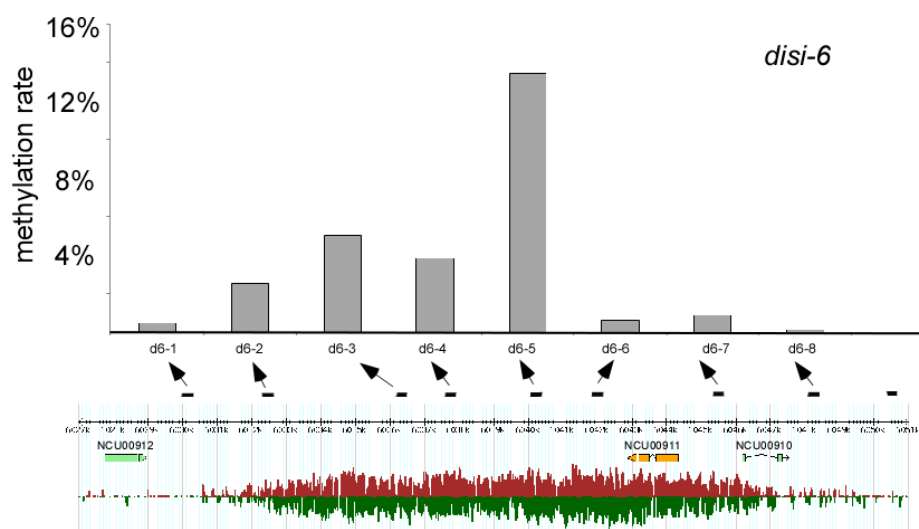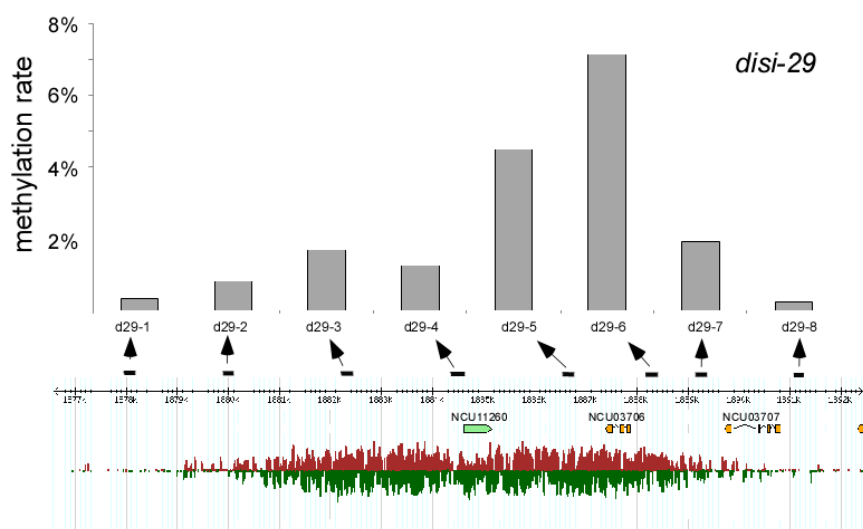

Figure S2

Supplement: Figure S2 — The MSP results for disi-6 and disi-29 loci determined by quantitative PCR. A wild type strain was used. (PDF) [file pgen.1003761.s002.pdf]

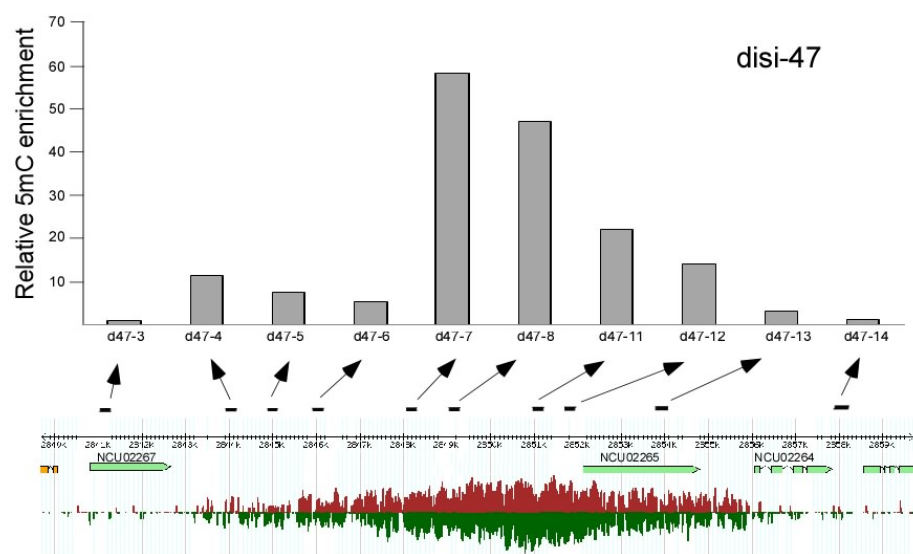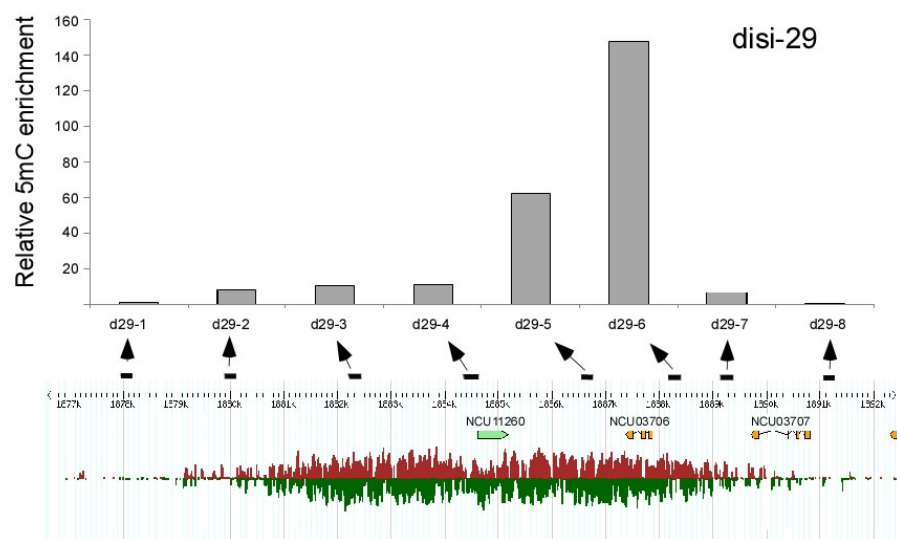

| Primer set              | al-1 | NCU06312 | ζ-η    |
|-------------------------|------|----------|--------|
| Relative 5mC enrichment | 1.02 | 1.76     | 201.58 |

Figure S3

Supplement: Figure S3 — MeDIP results for disi-47 and disi-29 loci of a wild type strain. The results of two negative controls (gene al-1 and NCU06312) and one positive control (ζ-η) are shown in the table. (PDF) [file pgen.1003761.s003.pdf]

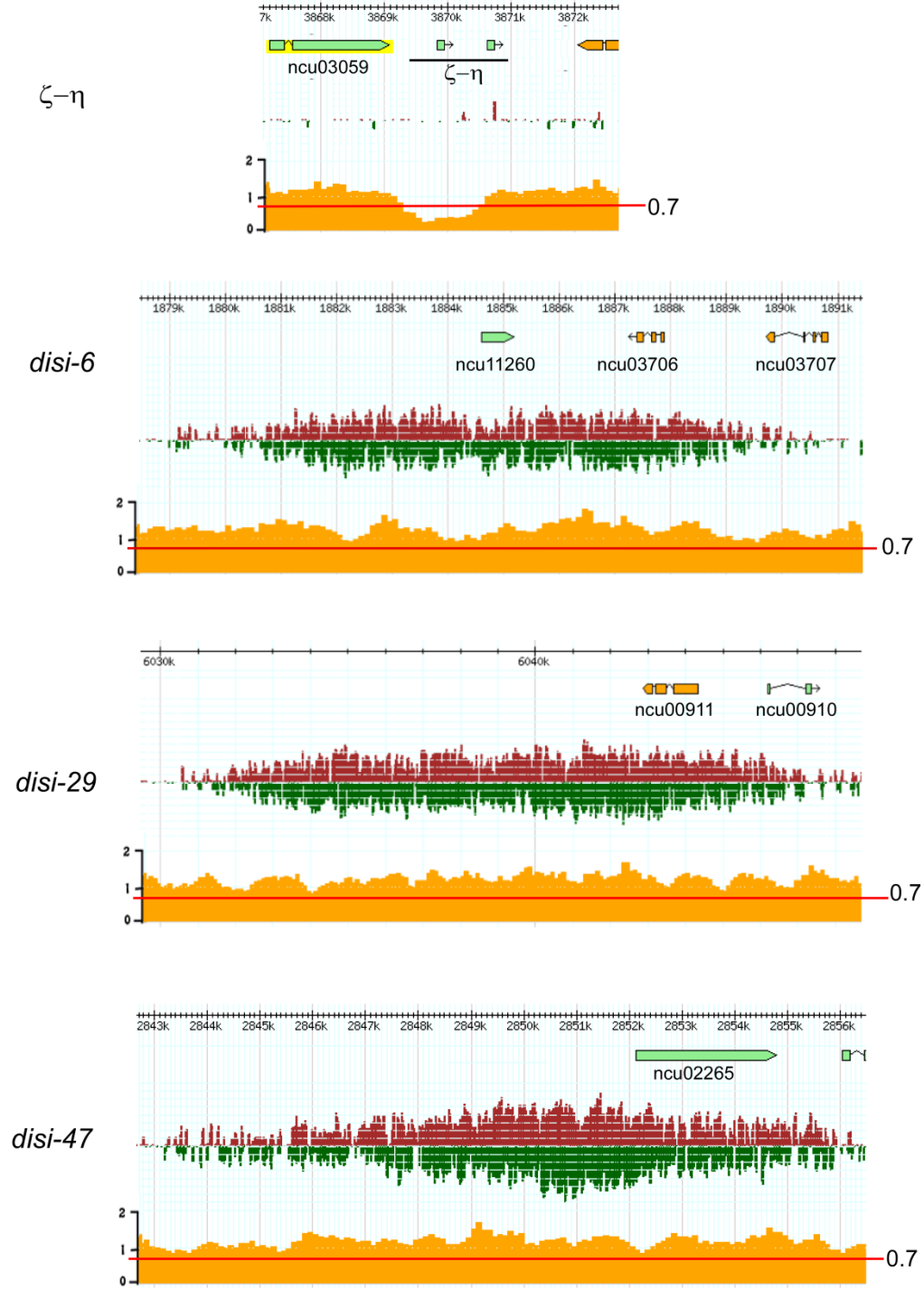

Figure S4

Supplement: Figure S4 — RIP indices in disiRNA loci. RIP indices were calculated with the number of CpA and TpG dinucleotides divided by number of ApC and GpT in a 500 bp window sliding every 100 bp across Neurospora genome [25]. The red lines indicate the RIP index threshold for RIP'd region (0.7). (PDF) [file pgen.1003761.s004.pdf]

*disi-47* (C=112)

BfuCI treated (73%)

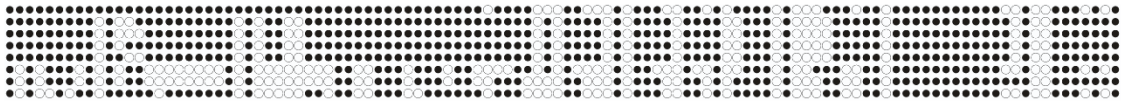

untreated (~0%)

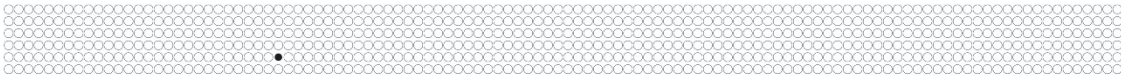

*disi-29* (C=78)

BfuCI treated (73%)

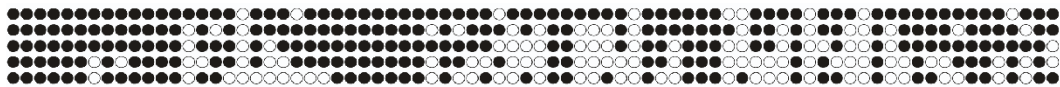

untreated (~0%)

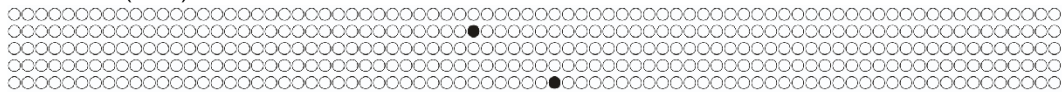

Figure S5

Supplement: Figure S5 — Bisulfite sequencing results for disi-47 and disi-29 loci. Experiments were performed with strategy 2 as described in Materials and Methods. Two aliquots of the wild-type genomic DNA, one treated with BfuCI and one untreated, were subjected to bisulfite conversion and nesting PCR (primer sequences in Table S2). PCR products were subcloned, sequenced and aligned. Each circle indicates one cytidine in sequenced regions. Opened and filled circles represent unmethylated and methylated C. (PDF) [file pgen.1003761.s005.pdf]

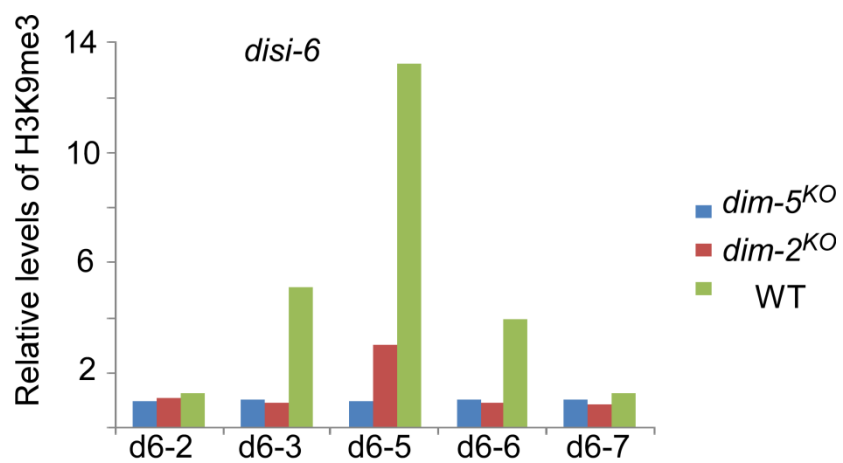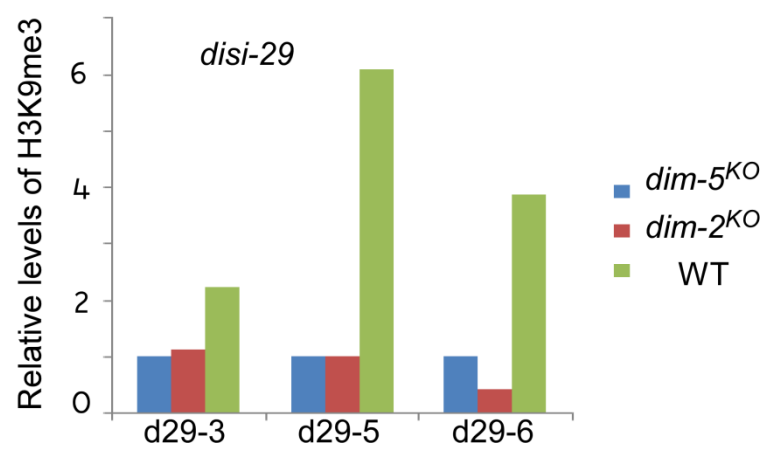

Figure S6

Supplement: Figure S6 — H3K9me3 ChIP results of disi-6 and disi-29 loci. For each primer set, the ChIP results of dim-5KO strain were set as 1. The primer set at am locus was used as loading control. (PDF) [file pgen.1003761.s006.pdf]

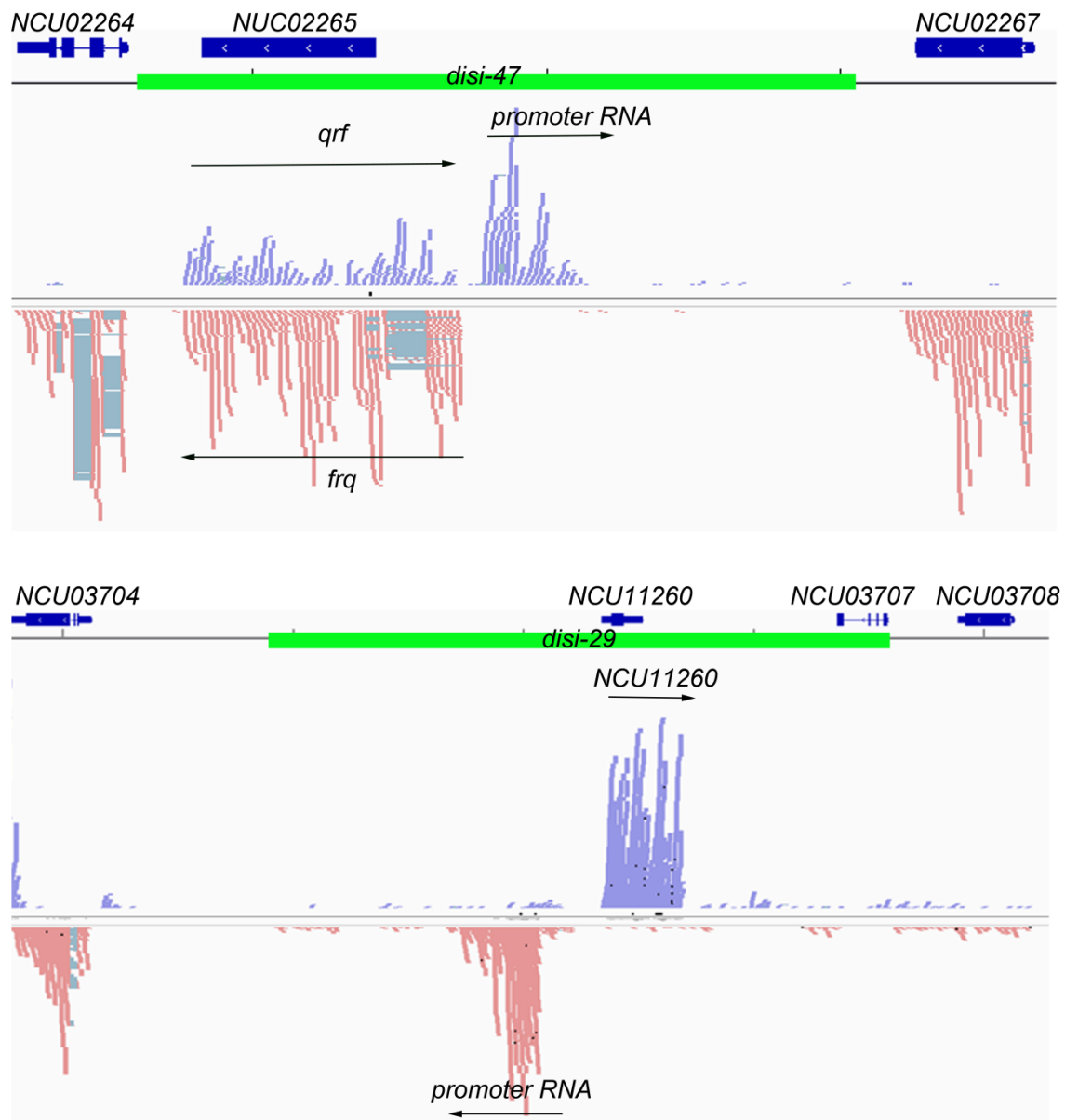

Figure S7

Supplement: Figure S7 — Strand-specific mRNA high throughput sequencing results of the disi-47 and disi-29 loci of wild type strain. Blue and red dots represent the reads that match Watson strand and Crick strand, respectively. The directionality of gene and promoter transcripts is indicated with arrows. The green bars indicate the disiRNA loci on Neurospora genome. The mRNA sequencing sample and data analyses were prepared as previous described [30]. (PDF) [file pgen.1003761.s007.pdf]

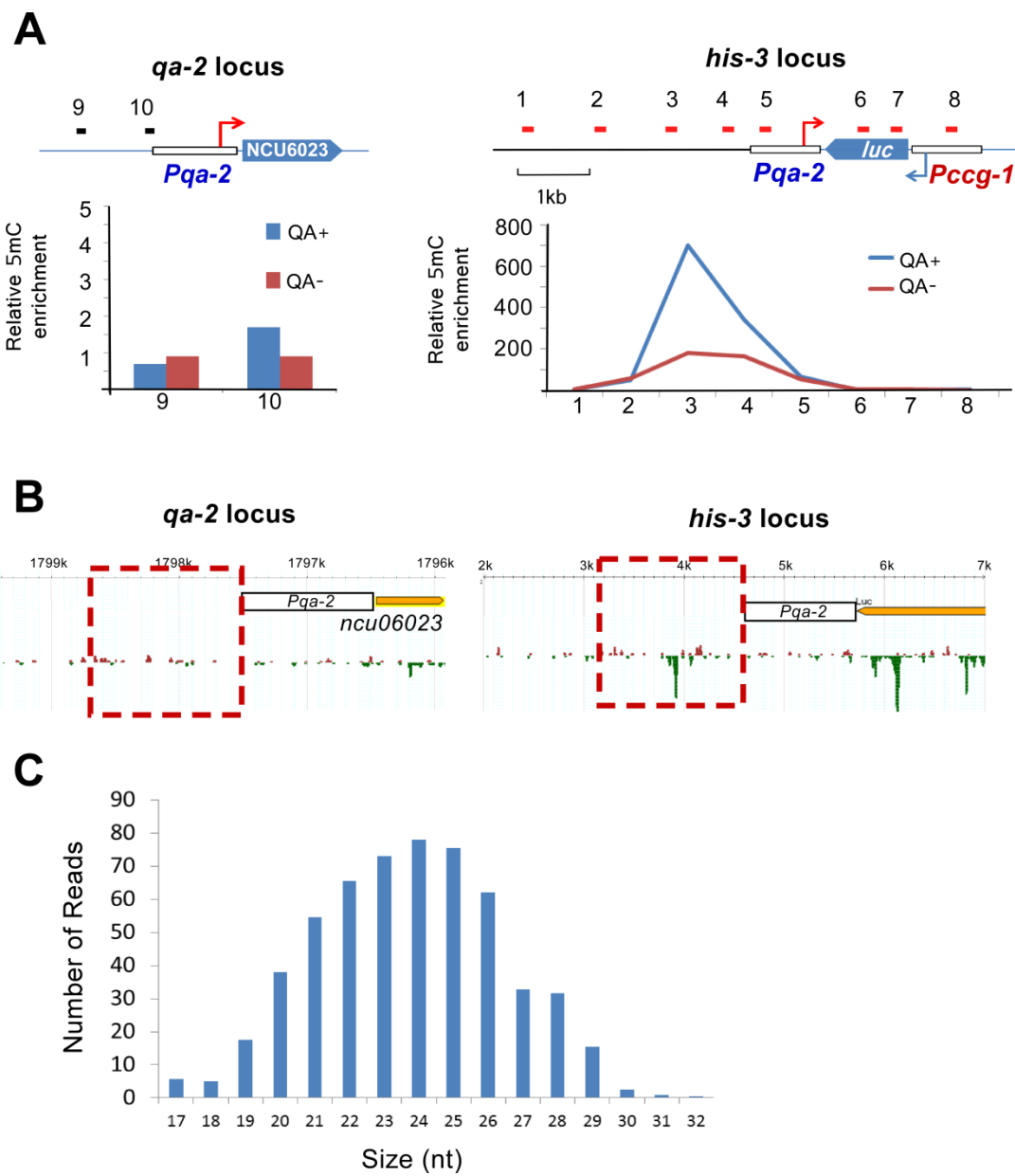

Figure S8

Supplement: Figure S8 — The DNA methylation is induced by convergent transcription and is correlated with disiRNA production. (A) An independent repeat for testing the DNA methylation triggered by convergent transcription. The experimental procedure was performed as described in Figure 5 except that a different Neurospora transformant was used. (B) The sRNA distribution at the endogenous qa-2 locus and recombinant his-3 locus. The result was from the dicerDKO strain harboring Pqa-2:cul:1-gccP construct upon QA induction. The red dashed boxes indicate the region upstream of the qa-2 promoter. (C) the size distribution of sRNA at the his-3 locus. (PDF) [file pgen.1003761.s008.pdf]
