# Supplementary material for: Convergent Transcription Induces Dynamic DNA Methylation at disiRNA Loci
Source: PLoS Genet. 2013 Sep 5;9(9):e1003761. doi: 10.1371/journal.pgen.1003761 (PMC3764098; doi:10.1371/journal.pgen.1003761)
Supplement: Table S1 — Sequences of primer sets used for detecting methylation in disiRNA loci. The approximate locations of these primer sets are indicated in Figure S1. (PDF) [file pgen.1003761.s009.pdf]

Table S1:

qPCR primer pairs for disiRNA-6 locus	
d6-1f	CGATTTGACGGCATTACTGGA
d6-1r	AGGCTCGTCAACGGAGTCTTC
d6-2f	GCCGTTTTCTCAACGTCTGT
d6-2r	GGCAAGGATGGCCTATGTTA
d6-3f	CATCACCACTTCGTCAGCAGAG
d6-3r	AGCCTGACTGCTGTACGAAAGG
d6-4f	CAGCGCCAAGTTTGACAGAA
d6-4r	CTGGACCACAGCATTCCTGTT
d6-5f	GATTGTCAAGCCGTTCTCGT
d6-5r	CCTGTGTTTCCGGATGAAGT
d6-6f	AATCGACTTCTCCCAGCTCGT
d6-6r	CCGTACTTTATCCAGGTGAGGC
d6-7f	GCGTGTAGCACTCCAACTGA
d6-7r	ATCTCTGAAGGCAGGCAGTG
d6-8f	CCGTGTGTGAAAGAACCCAGA
d6-8r	GTGCGAAGCTAGATGGTGTGG
d6-9f	ATTGTGGAGGATGGGGATAGG
d6-9r	CTGTTTGGTGCGTGTCTTCCT
qPCR primer pairs for disiRNA-29 locus	
d29-1f	CCTCGGATTTCTTTCACCTCTCTT
d29-1r	TACCTAGGCTGGGGCTCCTTTT
d29-2f	TGCACGCTATGGGAGTGTGT
d29-2r	CGCCGGAGAAAGATTGTGAT
d29-3f	AAAACATGTGGACGGTACGC
d29-3r	GGAAGGTCCAGACTGACGAC
d29-4f	GGACGCGGGTACATTACAAA
d29-4r	GCTGGTGCTTGCTGGTACTT
d29-5f	TCCCTATGGGCTGTTCTCAG
d29-5r	CTTTGCGGTAGCTTCTCCAC
d29-6f	GATAGGGGACGATGGGTGTA
d29-6r	CGTTCAAGCATGAAGGGTCT
d29-7f	AGTTACGGTCACCTTTGGTATCGAG
d29-7r	AGCCCAAAGTGCCTCATGTCA
d29-8f	ATTATGTGTCGGCTTCTGTGCG
d29-8r	TTTTGGAGCAAGCCAACCCT
disiRNA-47 primer pairs for disiRNA-47 locus	
d47-1f	GATAGAGAGAAACAATGGCTGCTTC
d47-1r	GGGGGGAGAAACTCACCAATTA
d47-2f	GACTCCAACGAGCTTGTCATCA
d47-2r	CGGCACCAAAATTGCTGTC
d47-3f	CAGAACCAACTTGGCGGTGAT
d47-3r	TTGACCTTGGTCGGGCAAA
d47-4f	ACCAATGGATGGATGGCCTAC
d47-4r	TTAGCCTAACGTGACGCATGC
d47-5f	CAGTGAAAAAGAGGCTCGCTG
d47-5r	CACGCCGAATAGAATCCAGAG

d47-6f	TCCTGTCTCGTTGCTTTCTCC
d47-6r	CAGGCGCTGATTAACGTGTTC
d47-7f	CCCCGTGTTTCTCCCAAGTAA
d47-7r	TGGGAATGGCTTGTCACACA
d47-8f	TGTGGGACGTTGCTCGATAG
d47-8r	TCTCGCTCGGTCATTGATTG
d47-9f	CTACACAGAAACACACCCGCA
d47-9r	AAAGTACACGACTACCCCGAGG
d47-10f	CTACACAGAAACACACCCGCA
d47-10r	CCCTGGGGTTTCCTTTGGA
d47-11f	TCCAAAGGAAACCCCAGGG
d47-11r	GGTGGATGGGGAAAAAAAGGA
d47-12f	TGTGTCGAGATTCCCTCCTC
d47-12r	CTCTCCCGCTCATGTTTGTT
d47-13f	GTTCCGCCAAGTTCGGTTT
d47-13r	CGAAAGAACCGTTCCTCCAA
d47-14f	AAGCGGTTTGTCGTTTTGGA
d47-14r	CCGGGCGGGAAATATAAAAA
d47-15f	CGCTTCCCAAATTGACCAAA
d47-15r	GGCGCCTACCTTTCTGGTTC
d47-16f	ACGCAACCCTCTCCCTTCTC
d47-16r	ATTCCGAAGACGGCCGATAC
qPCR primer pairs for other disiRNA loci	
disiRNA-23f	CTTCATCGCACTCAACGAAA
disiRNA-23r	GCTTCTGGTACATGCCACCT
disiRNA-35f	AATATCGAGTTTGGGCAACG
disiRNA-35r	CTCCCTGGTTCTCAGCAGAC
disiRNA-42f	TCACCGACGAATTTACCATCC
disiRNA-42r	GTTGAGATCGCCAACTTGAGG
disiRNA-48f	ATGTTCTGGTTCTTCTCTGCCG
disiRNA-48r	TCAACCAACCACCATCACTCC
disiRNA-49f	GTGGTGCCTCTACTTGTTCGGT
disiRNA-49r	CGCCTTTATGAACGGAATGG
disiRNA-8f	CTGGGAACACCACTGGAACA
disiRNA-8r	ATTGGTGAGGTGGGATCCTG
disiRNA-9f	GGCACCTCGTGGTTGTTGTA
disiRNA-9r	CTCCAGCAATTGAGCGTCTG
disiRNA-12f	GGTCAAGGTGGGTTTGAAGG
disiRNA-12r	CAGCAAGTCGCAGGCTACC
disiRNA-22f	TGCCAGTTGTACCGGTGAAG
disiRNA-22r	AGTCTTGGCCCAGAGCAGAC
disiRNA-28f	GGCCAGATGGATCCTACGAC
disiRNA-28r	GGGGTCGTCTGTGAAAGGAG
disiRNA-34f	CACTCTGGCCTTTTCCTTCCT
disiRNA-34r	AGCTCGACGTCAACCAGAGAC
disiRNA-39f	TGACAACTGAGGGCACACAT
disiRNA-39r	GCCGTAAAGCTCCATAGCTG
disiRNA-42f	GTTGAGATCGCCAACTTGAGG
disiRNA-42r	TCACCGACGAATTTACCATCC

disiRNA-50f	TATCGCGACTTCCCTCCTTC
disiRNA-50r	TGAACGCTCATTTCCCGTTA
**control primer pairs**	
am f	CGGTTACCGTGTCCAGTTCA
am r	CTAGAGACGCCGAGTCAGCA
Al-1 f	GCGATACCACGACAACACCA
Al-1 r	GGACCGTTCCGGAGAGAAAC
113-114 f	CAAAGAAGTCATGGAGCCCTCT
113-114 r	CGAGCATCACAAAATGGTCG
ncu06312f	CATGACTTGCCGCCAAGCT
ncu06312r	ATGTTCGCGGAGGTCCCTTT
ζ-η f	ACACTTAGGATTCGCTAATCGTC
ζ-η r	GTACGATCCTATCGGCTTAC
