# Supplementary material for: Convergent Transcription Induces Dynamic DNA Methylation at disiRNA Loci
Source: PLoS Genet. 2013 Sep 5;9(9):e1003761. doi: 10.1371/journal.pgen.1003761 (PMC3764098; doi:10.1371/journal.pgen.1003761)
Supplement: Table S2 — Primers used for preparing DNA probes for Southern blotting. (PDF) [file pgen.1003761.s010.pdf]

Table S2:

primer pairs for Southern blotting probes	
am f	CGGTTACCGTGTCCAGTTCA
am 3r	GCAAGGAAGAGCGATGTCAA
d29-5f	TCCCTATGGGCTGTTCTCAG
d29-6r	CGTTCAAGCATGAAGGGTCT
d47-8f	TGTGGGACGTTGCTCGATAG
d47-11r	GGTGGATGGGGAAAAAAAGGA
ζ-η probe r	AAA CCA TRT RCC CCA AAC CAC TCT
ζ-η probe f	GTT ATT GTA TAT YGA TAG GGA GAG AYY GG
