# Supplementary material for: Convergent Transcription Induces Dynamic DNA Methylation at disiRNA Loci
Source: PLoS Genet. 2013 Sep 5;9(9):e1003761. doi: 10.1371/journal.pgen.1003761 (PMC3764098; doi:10.1371/journal.pgen.1003761)
Supplement: Table S3 — Primers used for bisulfite sequencing of disiRNA loci. For each case, f1 and r1 were used for first round of PCR. The PCR product then was used to perform second round nesting PCR with f2 and r2. The am and al-1 were used as negative controls and ζ-η as positive control. (PDF) [file pgen.1003761.s011.pdf]

Table S3:

disiRNA-6	
ds6(+)f1	GGT GTT GYA TTC TGG GAA AGY TTG
ds6(+)f2	GGA TTT TGA GGG YAG YAG AYG GAG
ds6(+)r2	CTT AAC CCT TTT CTC TTA RAC CTR CRT C
ds6(+)r1	CAA CCC CAC CTA CCT CTT CAT C
disiRNA-29	
d29(+)f1	GTA YGG AGT ATT GGY TGG YTG TG
d29(+)f2	GGT TGG YYG GTY GTT GTT GTA AG
d29(+)r1	CTC RAT ARC CTRACC CCT TTC TCC
d29(+)r2	CAT CAC CAC CAC ATC CRT CRA C
disiRNA-47	
d47(-)f1	AAC TTT TCR ATC CRC TCR ATC CCC T
d47(-)r1	GGT ATT GYG ATA TGG GYA GAG TTG G
d47(-)f2	CTT RCC CTC CCT CCC CTT TCC AC
d47(-)r2	GTY YAG TTG AGG GTA TAG TGT TGG AAG
control primers: am locus	
am(+)f1	GTA GGT TTA TAA GGG TAY GTY TGA GAG A
am(+)f2	GTG TTG TTT GGG AGG AYG AYA AYG
am(+)r2	CCT TAC CAA TAA RRA CAC CCT CRA AAC
am(+)r1	CTC AAC CTC CTC CTT RRA AAC CTCA
control primers: al-1 gene	
al-1(+)f1	GTG YAG AGA GAA GGA GYG AYG ATA AG
al-1(+)f2	GAA GGT YGA GAT YGA AAA GTG GGA AG
al-1(+)r2	CTT RAC RCC CAT CCT CTC TCC AA
al-1(+)r1	CAC CTC RCC RTT CTC AAR CTR RAC AC
control primers: ζ-η	
ζ-η (+)f1	GAA GAA AGG AAG AAA AYA AAG AGA AAY YGA
ζ-η (+)f2	GTT ATT GTA TAT YGA TAG GGA GAG AYY GG
ζ-η (+)r1	CTA TCT CRC CCA ART CRA ARC CTC
ζ-η (+)r2	CCC TCC RCT AAC CCT TAT TAC TAT TAC
