# Supplementary material for: Convergent Transcription Induces Dynamic DNA Methylation at disiRNA Loci
Source: PLoS Genet. 2013 Sep 5;9(9):e1003761. doi: 10.1371/journal.pgen.1003761 (PMC3764098; doi:10.1371/journal.pgen.1003761)
Supplement: Table S4 — Primer sets used for strains with artificial convergent transcription and control strains. (PDF) [file pgen.1003761.s012.pdf]

Table S4:

qPCR primers	
1f	TCATGAACGCCGGTAGTGTC
1r	TCTGTGAAGCGGGGCTATTT
2f	GGGAGTGTGGGAAATGGTGT
2r	AACTATGCGGCATCAGAGCA
3f	GCATCTTACGGATGGCATGA
3r	CGTCGTTTGGTATGGCTTCA
4f	TTTCGCCACCTCTGACTTGA
4r	CAGGGGATAACGCAGGAAAG
5f	AACATCCGTGCAATGCAGAC
5r	AACCGTTGGAAAGCGAAGAA
6f	GATCATGTCCGGCTACGTCA
6r	GTGCTGGAGGAGGATGGACT
7f	TCGGGCATCTTCACAACAAC
7r	GAGGGAGTTCTCGGAGCAGA
8f	CCCTTAATCGGCAATCGAGA
8r	ATCCGCGTTGGGATTATGAC
9f	TGTGTGCTTCATTCCCTCCTT
9r	TGGCATTGTTGTTGCTGAATC
10f	CGACCCTGGATCTCGCTTAC
10r	CTAAGCCCGTCCCATTCTTG
bisulfite primers	
qa(-) 1f	TCT CAT RAC CAA AAT CCC TTA AC
qa(-) 2f	TCC ACT AAA CAT CAR ACC CCR TA
qa(-) 1r	GGG AAG TGT GGT GTT TTY TYA ATG
qa(-) 2r	GTT TGT TTT AAG YTG GGY TGT GTG
